# The Pattern of Cytokines, Chemokines, and Growth Factors of the Maxillary and Mandibular Periosteum After Exposure to Titanium Fixations—Ti6Al4V

**DOI:** 10.3390/jcm13237064

**Published:** 2024-11-22

**Authors:** Bożena Antonowicz, Mateusz Maciejczyk, Jan Borys, Kamila Łukaszuk, Sara Zięba, Edyta Gołaś, Małgorzata Żendzian-Piotrowska, Anna Zalewska

**Affiliations:** 1 Department of Dental Surgery, Medical University in Bialystok, 15-089 Białystok, Poland; 2 Department of Hygiene, Epidemiology and Ergonomics, Medical University of Bialystok, 15-089 Białystok, Poland; 3 Department of Maxillofacial and Plastic Surgery, Medical University of Bialystok, 15-089 Białystok, Poland; 4 Department of Restorative Dentistry, Medical University of Bialystok, 15-089 Białystok, Poland; 5 Independent Laboratory of Experimental Dentistry, Medical University of Bialystok, 15-089 Białystok, Poland

**Keywords:** cytokines, chemokines, growth factors, dentofacial deformities, titanium miniplates and screws

## Abstract

**Objectives:** Titanium miniplates and screws are commonly used in the surgical management of dentofacial deformities. Despite the opinion of the biocompatibility of these bone fixations, some patients experience symptoms of chronic inflammation around titanium implants even many years after their application. The aim of this study was to examine the levels of cytokines, chemokines, and growth factors released from the maxilla and mandible periosteum surrounding titanium fixations 11 months after the implantation procedure. **Methods:** From the study group (n = 20) consisting of patients with maxillofacial defects who underwent bimaxillary osteotomy, fragments of the periosteum of the maxilla and mandible adjacent to the titanium miniplates and screws were taken during routine bone fixation removal procedures. From the control group subjects (n = 20), fragments of healthy maxillary and mandibular periosteum were taken prior to surgical treatment of dentofacial deformities. The examination of cytokines, chemokines, and growth factors levels released from the periosteum of jaws was performed using the Bio-Plex Pro Human Cytokine Screening Panel (48-Plex). **Results:** The study group was characterized by a significant increase in the concentration of most of the tested-for proinflammatory cytokines/chemokines/growth factors compared to the control group, with greater amounts of inflammatory factors released from the periosteum covering the titanium implants in the mandible than from the periosteal cells surrounding the titanium implants in the maxilla. **Conclusions:** Prolonged exposure to titanium miniplates and screws leads to a disturbance of immune homeostasis in the periosteal cells of the maxilla and mandible. The data obtained indicate the need to remove fixations after the bone fragments have healed.

## 1. Introduction

Modern surgical methods allow for the restoration of function and aesthetics, which affects both the physical and mental health of patients who have undergone the treatment of facial injuries and orthognathic surgery [1,2,3,4]. Titanium and its alloys, from which implants, screws, stents, and many other biomedical implants are made, are most commonly used in surgical procedures [1,4]. This common use of products made from titanium alloys is due to its low toxicity, slight solubility under physiological pH conditions, and relatively low reactivity with biomolecules [4]. The latter characteristic classifies titanium and its alloys as biocompatible materials. However, a biocompatible material should not cause inflammation or allergies. The related literature more and more often reports on high cytotoxicity and, consequently, the harmful effects of titanium on tissues and organs [1,2,3,4,5]. In order to reduce the harmful impact of titanium and its alloys on surrounding tissues, the surface of titanium implants is coated with a passive layer of titanium dioxide (TiO_2_), formed through a process called anodization. Although the passive layer is intended to reduce the corrosion potential of the alloy, many patients still experience the phenomenon known as metallosis [2,4,5,6,7]. Metallosis is the process of metallic particle deposition around the implant due to the degradation processes that the metal or its alloys are subject to [2,4,5,6,7]. That phenomenon is facilitated by mechanical friction and chemical influences that lead to damage or weakening of the TiO_2_ layer [4]. That process results in the corrosion of the alloy and the deposition of metallic particles at the implantation site over the course of the “use” of the titanium implants [5]. It is worth noting that implants exposed to large forces and stresses, such as those in the mandible, are particularly susceptible to metallosis [1,4,5]. The mere presence of titanium bone implants in the form of miniplates and screws, and even more so the products of their wear and tear, disturbs the body’s immunological processes and accounts for oxidative stress as well as inflammation [1,2,3,4]. It may lead to the necessity of further surgical procedures arising from the need to remove titanium implants, and, on the other hand, it may cause complications in distant organs [1,2,3,4,8].

It should be noted that stimulation of the immune system is essential in the process of healing and osteointegration [9]. Immediately after the implantation of a titanium implant, the so-called acute phase of inflammation develops, during which immunocompetent cells, mainly polymorphonuclear leukocytes (PMNs), migrate from blood vessels toward the implant, initiating a complex wound healing process. Other cells, including mononuclear cells (monocytes) that transform into macrophages, are subsequently activated, initiating the chronic phase of inflammation, which should lead to bone formation and osteointegration around the titanium implants (dental implants as well as miniplates and screws) [9]. Unfortunately, inflammatory symptoms in the vicinity of unremoved fixations have been reported by patients and clinically observed both after several months and after years following osteosynthesis of the maxilla or mandible [1]. Therefore, the question arises whether leaving the implants permanently is safe for the patient. The long-term (beyond the period of fracture healing) impact of titanium miniplates and screws on the immunological processes in the surrounding tissues is unknown.

In this study, the aim has been to examine cytokines, chemokines, and growth factors released from the periosteum of maxilla and mandible surrounding titanium fixations after 11 months following the implantation procedure. As clinical observations show inflammatory complications following the implantation of titanium miniplates and screws are more frequent in the mandible than in the maxilla. We have also formulated a research hypothesis that inflammatory processes in the periosteum surrounding titanium fixations are more pronounced in the mandible than in the maxilla. Therefore, our aim has been to assess the differences in the stimulation of the immune system in the periosteal cells of the maxilla and mandible, thereby enhancing our understanding of that clinical phenomenon.

## 2. Materials and Methods

### 2.1. Ethical Issues

The research was conducted in accordance with the Declaration of Helsinki—1964 (with modification October 2013, Fortaleza, Brasil). All patients were informed about the manner of this study, the collection of research material (blood and periosteum), and agreed in writing to participate in the experiment. The protocol of this study was approved by the Bioethics Committee of the Medical University of Bialystok (APK.002.71.2023, APK.002.72.2023; from 19 January 2023 till 30 December 2030).

### 2.2. Patients

The 40 patients were treated at the Department of Maxillofacial and Plastic Surgery at the Medical University of Bialystok, Poland, from 1 March 2023 to 1 February 2024, because of dentofacial deformities of class II and III of the Angle classification.

The study group included of 10 women and 10 men, aged 21 to 30, who underwent osteotomy of the maxilla and mandible with fixation of the osteotomy fragments using titanium miniplates and screws. Titanium fixations were made of titanium alloy Ti6Al4V, produced by ChM Lewickie Sp. z o. o., Lewickie, Poland.

All patients in the study group underwent Le Fort I maxillary osteotomy and Obwegeser–Dal Pont mandibular osteotomy. The surgical procedures applied were the same for all patients.

The control group, selected by sex and age to match the study group, consisted of 20 generally healthy patients (10 women and 10 men), aged 21 to 30, operated on due to skeletal deformities, before surgical treatment.

Women participating in the experiment were always in the first phase of their menstrual cycle when collecting the material.

The titanium bone fixations were surgically removed under general anesthesia 11 months after bimaxillary osteotomy, once bone fusion was achieved. Bone union was assessed by control radiographs taken routinely before removal of titanium miniplates and screws, as well as intraoperatively. Radiological diagnostics included teleroentgenogram in the PA (Posterior–Anterior) projection and cephalometry in the lateral projection (Cranex 3D, Soredex, Tuusula, Finland). All surgical procedures were performed under general anesthesia by an experienced surgeon (J.B.), a specialist in maxillofacial surgery, and his team of assistants.

A month before the procedure, immediately after the procedure, and until the removal of the titanium bone fixations, patients followed a balanced diet (2200 kcal: 55% carbohydrates, 30% fat, and 15% protein), designed by a dietitian and remained under his supervision. During their stay at the Department of Maxillofacial and Plastic Surgery, the patients were thoroughly examined before and after surgery, as well as after hospitalization. The participants underwent follow-up examinations to thoroughly assess their general and local condition.

#### 2.2.1. Inclusion Criteria to Study Group

Generally healthy patients with dentofacial deformities of class II and III of the Angle classification who underwent jaws bone titanium fixations: four miniplates used in the maxilla and mandible (two 4–5 hole miniplates on the right and left side), fastened with four screws in each plate used for the surgical treatment of dentofacial deformities.

#### 2.2.2. Inclusion Criteria to Control Group

Generally healthy patients with dentofacial deformities of class II and III of the Angle classification before a surgical procedure using titanium bone fixations.

#### 2.2.3. Inclusion Criteria to Study and Control Group

Negative history of systemic diseases (inflammatory, metabolic, gastrointestinal, cardiological, neurological, rheumatological, nephrological, autoimmunological, focal, and oncological), eating disorders like bulimia or anorexia, and mental disorders.

Absence of concurrent or previous local disorders (dental pulpitis, gingivitis periodontitis, osteomyelitis, active odontogenic infection foci, and neoplasms).
No previous titanium dental and bone implants, joint prostheses, vascular clasps, or orthodontic screws.
No past or present use of permanent prosthetic restorations or removable dentures and orthodontic appliances.
Not treated for bone fractures.
Non-cigarette and e-cigarette smokers.
Non-drug users.
Non-alcohol drinkers.
Not taking any medicaments (antibiotics, nonsteroidal inflammatory drugs, hormones, corticosteroids, anti-epileptic drugs, anticoagulants, diuretics, or vitamins or dietary supplements).

#### 2.2.4. Exclusion Criteria to Study and Control Group

Another type of surgical procedure on the jaws.

Inflammatory complications and disturbances of the healing process of the jaws after osteotomy.

Other diseases, both local and general after surgery and course of treatment.

Poor oral hygiene, active caries, or a periapical process.

### 2.3. Material Collection

In the control group, small fragments of healthy periosteum (3 mm × 7 mm, 1 mm thick) of both the maxilla (Max Control) and the mandible (Man Control) were taken from the same patients during surgery, before performing osteotomy of the maxilla and mandible and before the inserting of titanium fixations. These fragments of periosteum covering the osteotomy segments are routinely removed before insertion of titanium miniplates and screws.

In the study group, gray discolored periosteum of the maxilla (Max Study) and mandible (Man Study) adjacent to the miniplates and screws (measuring 3 mm × 7 mm, 1 mm thick) was collected from the same patient during the routine bone fixation removal procedure. These periosteum pieces are usually removed and disposed of.

### 2.4. Preparation of Periosteum Homogenates

The tissues were removed, immediately frozen in liquid nitrogen, and stored at −80 °C until use. They were rinsed in ice-cold PBS (phosphate-buffered saline) (0.02 mol L^−1^, pH 7.0–7.2) to be cleaned from any remaining blood elements. Tissue samples were homogenized then diluted in PBS with a homogenizer (Omni TH, Omni International, Kennesaw, GA, USA) on ice and sonicated with an ultrasonic cell disrupter (1800 J per sample, 20 s × 3 on ice, UP 400S; Hielscher, Teltow, Germany) for further cell membrane breakdown. The homogenates were centrifuged for 5 min at 5000× *g*, 4 °C. Then, the supernatant was discarded and the remaining liquid material was assayed on the same day.

### 2.5. Measurement of Cytokines, Chemokines and Growth Factors

The concentrations of cytokines (IFN-α2—Interferon-alpha 2, IFN-γ—Interferon-gamma, IL-1α—Interleukin-1 alpha, IL-1β—Interleukin-1 beta, IL-1ra—Interleukin-1receptor antagonist, IL-2—Interleukin-2, IL-2Rα—Interleukin-2Receptor alpha, IL-3—Interleukin-3, IL-4—Interleukin-4, IL-5—Interleukin-5, IL-6—Interleukin-6, IL-7—Interleukin-7, IL-9—Interleukin-9, IL-10—Interleukin-10, IL-12(p70)—Interleukin-12(p70), IL-12(p40)—Interleukin-12(p40), IL-13—Interleukin-13, IL-15—Interleukin-15, IL-16—Interleukin-16, IL-17—Interleukin-17, IL-18—Interleukin-18, MIF—Macrophage Migration Inhibitory Factor, TNF-α—Tumor Necrosis Factor-alpha, TNF-β—Tumor Necrosis Factor-beta, and TRAIL—TNF-related apoptosis-inducing ligand), chemokines (CTACK—Cutaneous T Cell-Attracting Chemokine, GRO-α—Growth-Regulated Oncogene-alpha, IP-10—Interferon-Inducible Protein-10, LIF—Leukemia Inhibitory Factor, MCP-1—Monocyte Chemoattractant Protein-1, MCP-3—Monocyte Chemoattractant Protein-3, M-CSF—Macrophage Colony-Stimulating Factor, MIG—Monokine Induced by Gamma Interferon, MIP-1α—Macrophage Inflammatory Protein-1 alpha, MIP-1β—Macrophage Inflammatory Protein-1 beta, IL-8—Interleukin-8, SDF-1α—Stromal Cell-Derived Factor-1alpha, and RANTES—Regulated on Activation, Normal T Cell Expressed and Secreted), and growth factors (Basic FGF—Basic Fibroblast Growth Factor, HGF—Hepatocyte Growth Factor, β-NGF—Nerve Growth Factor-beta, PDGF-BB—Platelet Derived Growth Factor-BB, SCGF—β—Stem Cell Growth Factor-beta, VEGF—Vascular Endothelial Growth Factor, G-CSF—granulocyte colony stimulating factor, GM-CSF—granulocyte macrophage-colony stimulating factor, and SCF—Stem Cell Factor) were measured using the Bio-Plex Pro Human Cytokine Screening Panel, 48-Plex (#12007283, Bio-Rad Laboratories, Inc., Hercules, CA, USA). The Bio-Plex multiplex assay is based on the standard ELISA test, where antibodies directed against a specific biomarker are covalently linked to magnetic beads. The conjugated beads react with a sample containing the selected biomarker, followed by a series of washes to remove unbound protein. In the next step, a biotinylated detection antibody is added, forming a sandwich-type complex. The final complex is created by adding a streptavidin–phycoerythrin conjugate, and the result is read using a specialized plate reader (Bio-Plex 200). The concentrations of cytokines, chemokines, and growth factors were standardized to the total protein content, which was measured spectrophotometrically (Thermo Scientific PIERCE BCA Protein Assay; Rockford, IL, USA).

### 2.6. Statistical Analysis

The obtained data were analyzed with GraphPad Prism 10.2.0. statistical software for macOS (GraphPad Software, La Jolla, CA, USA) using the Shapiro–Wilk test to assess the normality of the distribution. The Mann–Whitney U-test was used to compare the groups; its results are shown in the bar plots as median (minimum–maximum) and quartiles. A *p*-value < 0.05 was considered statistically significant.

## 3. Results

### 3.1. Periosteum of Maxilla

**Figure 1:**
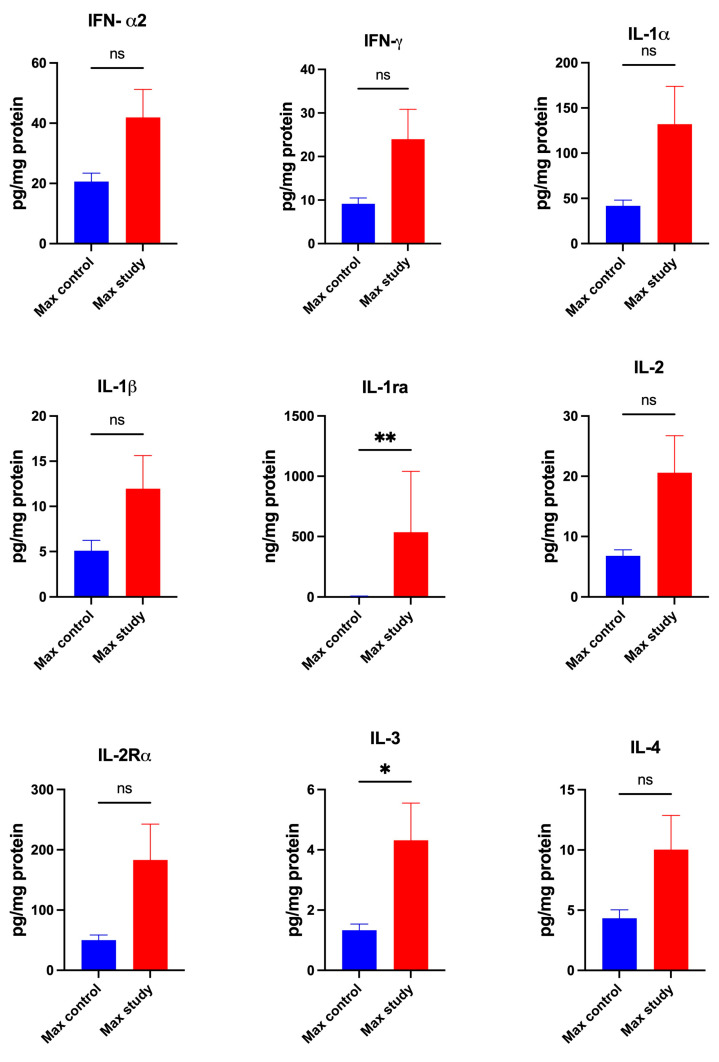
Concentrations of the cytokines in maxillary periosteum: IFN-α2—Interferon-alpha 2, IFN-γ—Interferon-gamma, IL-1α—Interleukin-1 alpha, IL-1β—Interleukin-1 beta, IL-1ra—Interleukin-1receptor antagonist, IL-2—Interleukin-2, IL-2Rα—Interleukin-2Receptor alpha, IL-3—Interleukin-3, IL-4—Interleukin-4, Max—maxilla, * *p* < 0.05, ** *p* < 0.005, ns—not significant.

**Figure 2:**
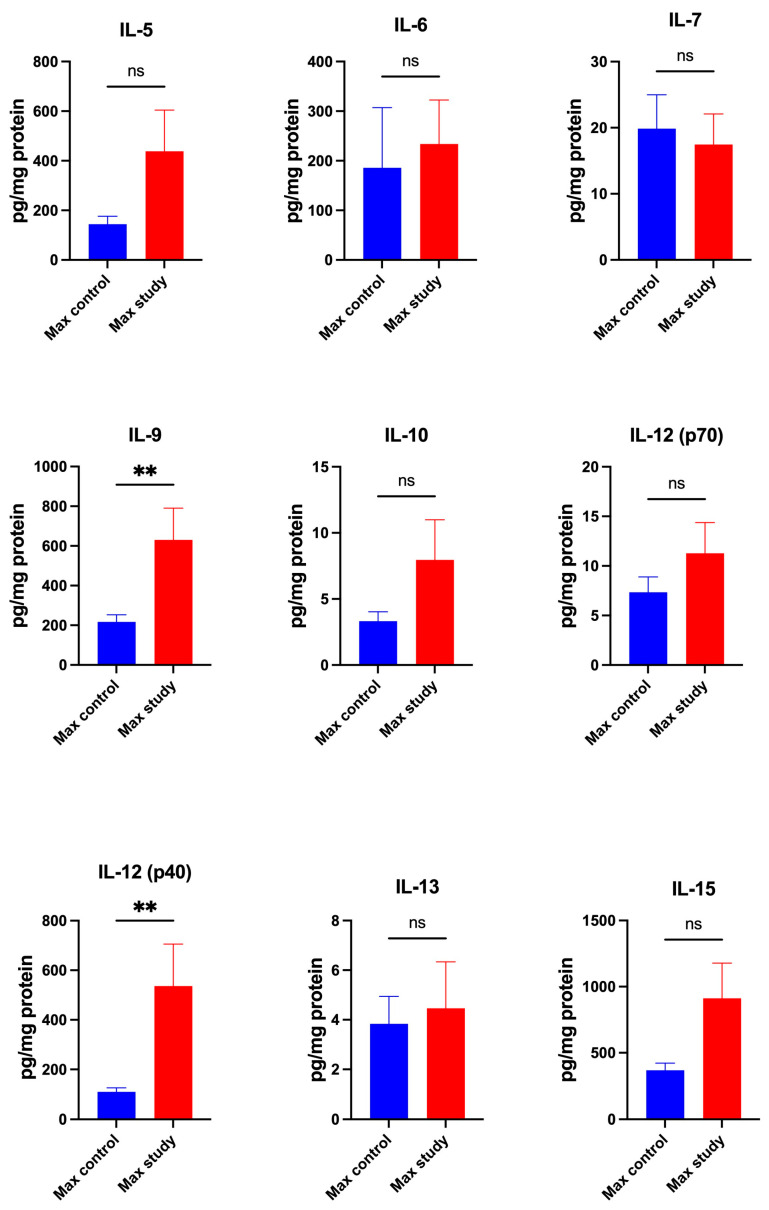
Concentrations of the cytokines in maxillary periosteum: IL-5—Interleukin-5, IL-6—Interleukin-6, IL-7—Interleukin-7, IL-9—Interleukin-9, IL-10—Interleukin-10, IL-12(p70)—Interleukin-12(p70), IL-12(p40)—Interleukin-12(p40), IL-13—Interleukin-13, IL-15—Interleukin-15, Max—maxilla, ** *p* < 0.005, ns—not significant.

**Figure 3:**
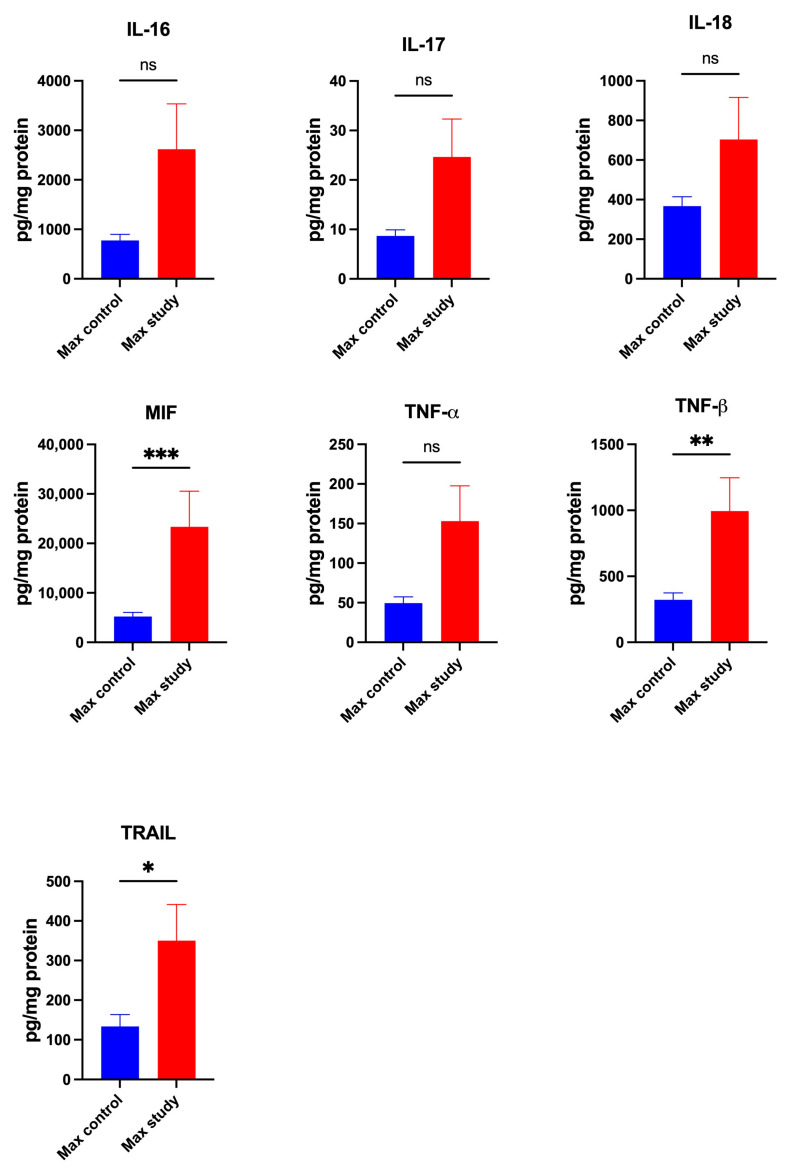
Concentrations of the cytokines in maxillary periosteum: IL-16—Interleukin-16, IL-17—Interleukin-17, IL-18—Interleukin-18, MIF—Macrophage Migration Inhibitory Factor, TNF-α—Tumor Necrosis Factor-alpha, TNF-β—Tumor Necrosis Factor-beta, TRAIL—TNF-related apoptosis-inducing ligand, Max—maxilla, * *p* < 0.05, ** *p* < 0.005, *** *p* < 0.0005, ns—not significant.

The concentrations of cytokines IL-1ra (*p* < 0.005) and IL-3 (*p* < 0.05) (Figure 1); IL-9 (*p* < 0.005) and IL-12(p40) (*p* < 0.005) (Figure 2); and MIF (*p* < 0.0005), TNF-β (*p* < 0.005), and TRAIL (*p* < 0.0005) were significantly higher in the maxillary periosteum of the study group compared to control (Figure 3).

The concentrations of: IFN-α2, IFN-γ, IL-1α, IL-1β, IL-2, IL-2Rα, IL-4, IL-5, IL-6, IL-7, IL-10, IL-12(p70), IL-13, IL-15, IL-16, IL-17, IL-18, and TNFα were similar in both groups (Figure 1, Figure 2 and Figure 3).

**Figure 4:**
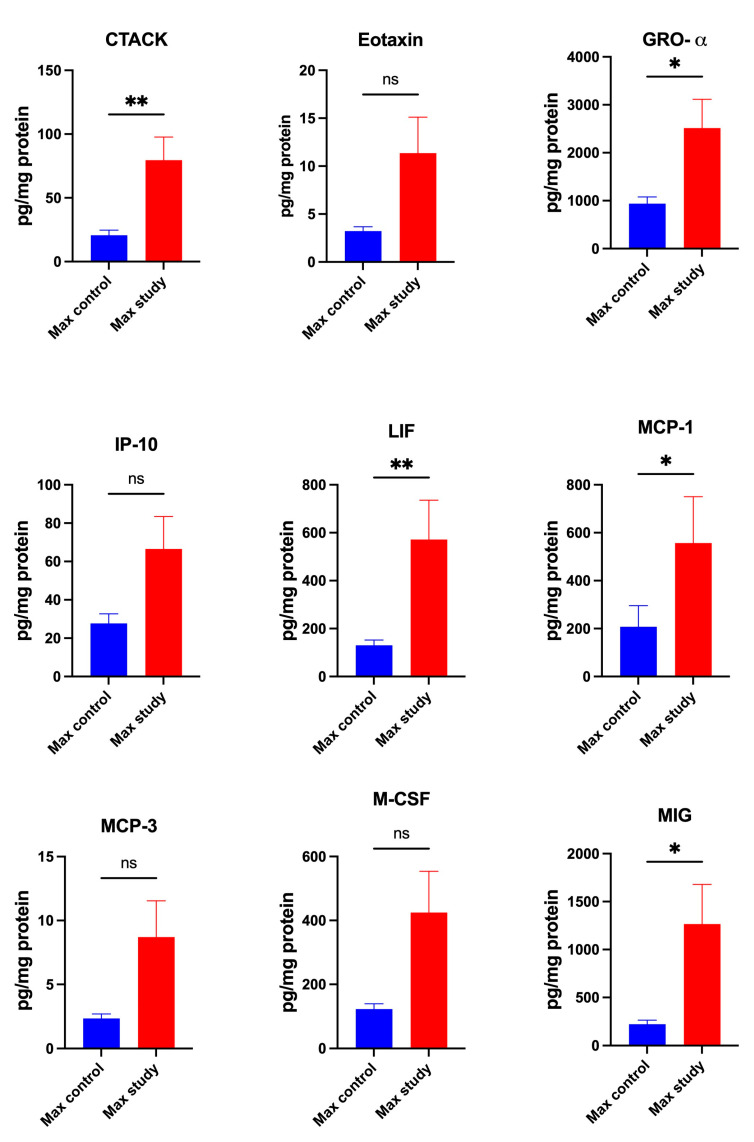
Concentrations of the chemokines in maxillary periosteum: CTACK—Cutaneous T Cell-Attracting Chemokine, GRO-α—Growth-Regulated Oncogene-alpha, IP-10—Interferon-Inducible Protein-10, LIF—Leukemia Inhibitory Factor, MCP-1—Monocyte Chemoattractant Protein-1, MCP-3—Monocyte Chemoattractant Protein-3, M-CSF—Macrophage Colony-Stimulating Factor, MIG—Monokine Induced by Gamma Interferon, Max—maxilla, * *p* < 0.05, ** *p* < 0.005, ns—not significant.

**Figure 5:**
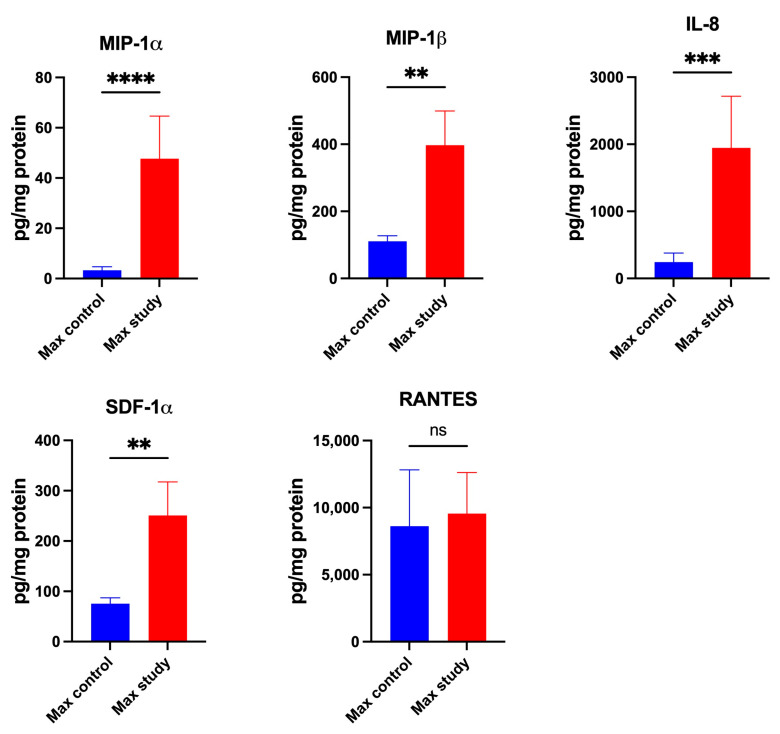
Concentrations of the chemokines in maxillary periosteum: MIP-1α—Macrophage Inflammatory Protein-1 alpha, MIP-1β—Macrophage Inflammatory Protein-1 beta, IL-8—Interleukin-8, SDF-1α—Stromal Cell-Derived Factor-1alpha, RANTES—Regulated on Activation, Normal T Cell Expressed and Secreted, Max—maxilla, ** *p* < 0.005, *** *p* < 0.0005, **** *p* < 0.00001, ns—not significant.

The concentrations of chemokines, CTACK (*p* < 0.005), GRO-α (*p* < 0.05), LIF (*p* < 0.005), MCP-1 (*p* < 0.05), and MIG (*p* < 0.05) (Figure 4) and MIP-1α (*p* < 0.0001), MIP 1-β (*p* < 0.005), IL-8 (*p* < 0.0005), and SDF-1α (*p* < 0.005) (Figure 5), were significantly higher in the maxillary periosteum of the patients in the study group than in the control.

No significant differences in the concentrations of Eotaxin, IP-10, MCP-3, M-CSF, and RANTES in the maxillary periosteum were observed in both groups (Figure 4 and Figure 5).

**Figure 6:**
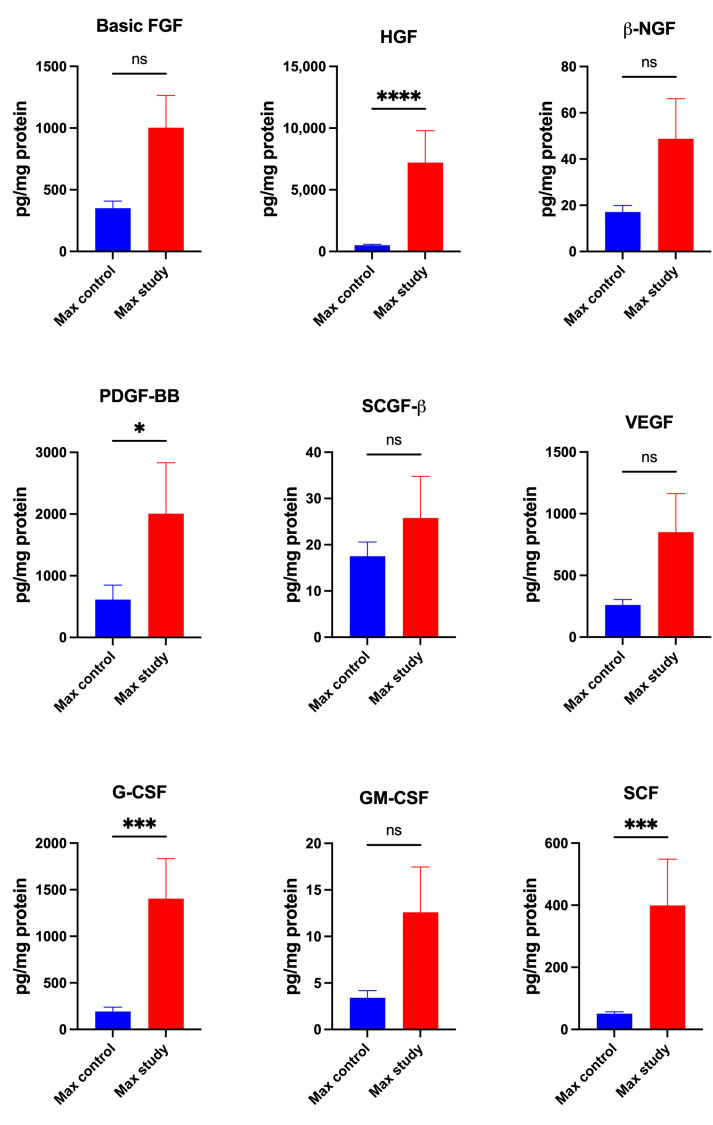
Concentrations of the growth factors in maxillary periosteum: Basic FGF—Basic Fibroblast Growth Factor, HGF—Hepatocyte Growth Factor, β-NGF—Nerve Growth Factor-beta, PDGF-BB—Platelet Derived Growth Factor-BB, SCGF-β—Stem Cell Growth Factor-beta, VEGF—Vascular Endothelial Growth Factor, G-CSF—granulocyte colony stimulating factor, GM-CSF—granulocyte macrophage-colony stimulating factor, SCF—Stem Cell Factor, Max—maxilla, * *p* < 0.05, *** *p* < 0.005, **** *p* < 0.00001, ns—not significant.

The experimental group patients were characterized by significantly higher concentrations of growth factors HGF (*p* < 0.0001), PDGF-β (*p* < 0.05), G-CSF (*p* < 0.0005), and SCF (*p* < 0.0005) in the periosteum of maxilla than control subjects (Figure 6).

The concentrations of Basic FGF, β-NGF, SCGF-β, VEGF, and GM-CSF were similar in both groups (Figure 6).

### 3.2. Periosteum of Mandible

**Figure 7:**
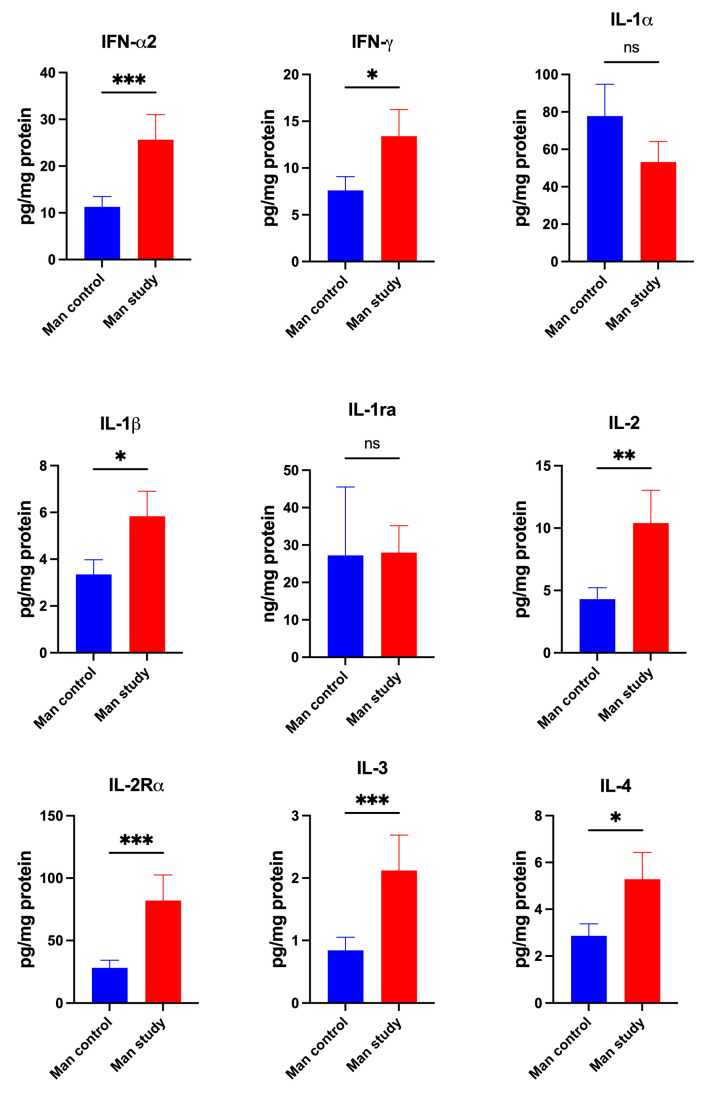
Concentrations of the cytokines in mandibular periosteum: IFN-α2—Interferon-alpha 2, IFN-γ—Interferon-gamma, IL-1α—Interleukin-1 alpha, IL-1β—Interleukin-1 beta, IL-1ra—Interleukin-1receptor antagonist, IL-2—Interleukin-2, IL-2Rα—Interleukin-2Receptor alpha, IL-3—Interleukin-3, IL-4—Interleukin-4, Man—mandible, * *p* < 0.05, ** *p* < 0.005, *** *p* < 0.0005, ns—not significant.

**Figure 8:**
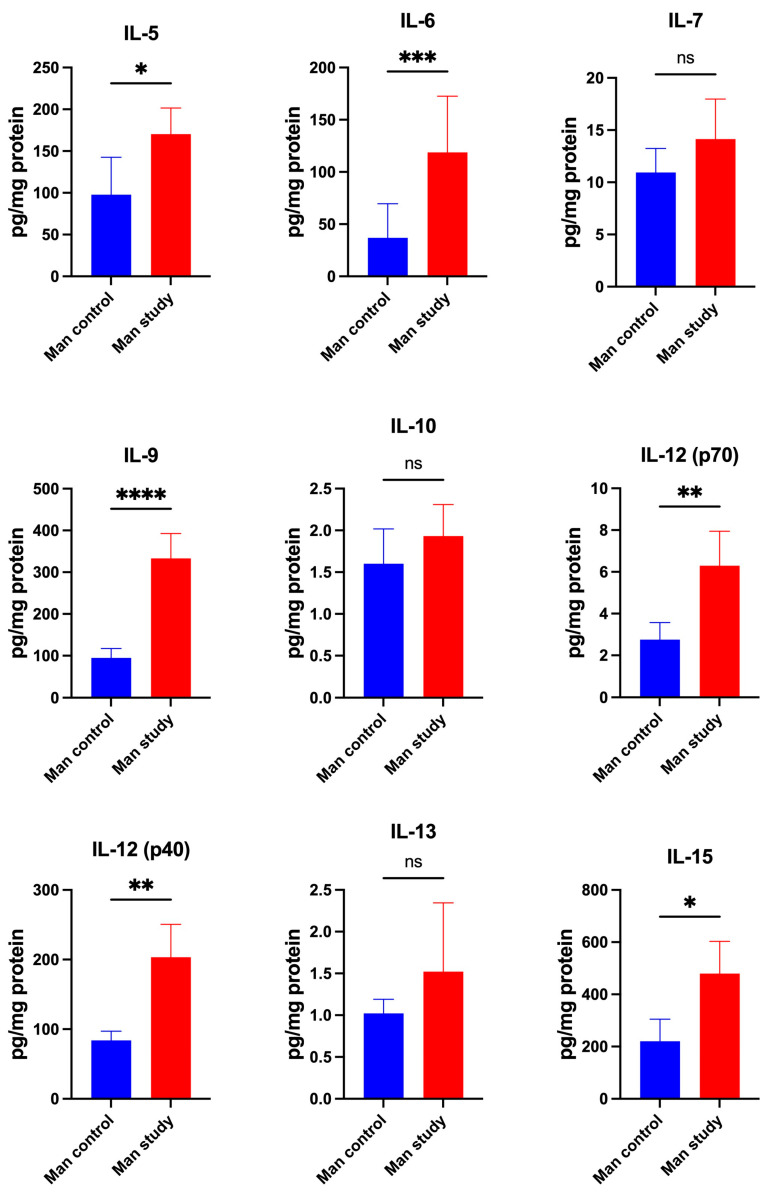
Concentrations of the cytokines in mandibular periosteum: IL-5—Interleukin-5, IL-6—Interleukin-6, IL-7—Interleukin-7, IL-9—Interleukin-9, IL-10—Interleukin-10, IL-12(p70)—Interleukin-12(p70), IL-12(p40)—Interleukin-12(p40), IL-13—Interleukin-13, IL-15—Interleukin-15, Man—mandible, * *p* < 0.05, ** *p* < 0.005, *** *p* < 0.0005, **** *p* < 0.0001, ns—not significant.

**Figure 9:**
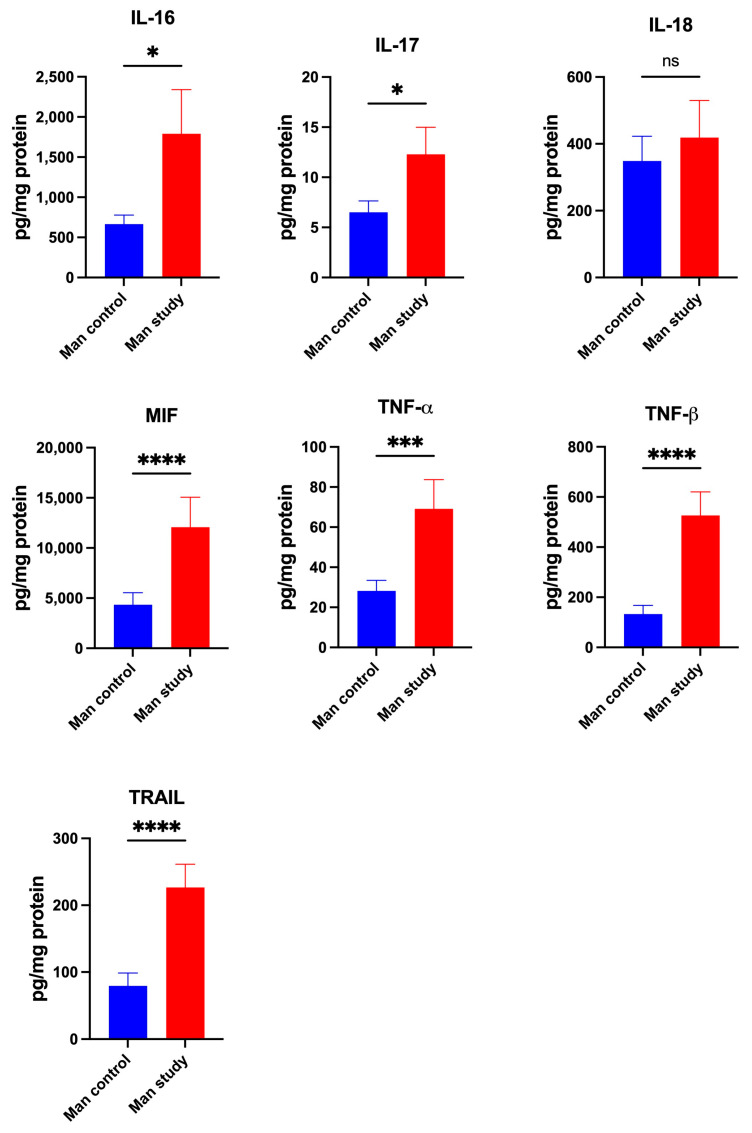
Concentrations of the cytokines in mandibular periosteum: IL-16—Interleukin-16, IL-17—Interleukin-17, IL-18—Interleukin-18, MIF—Macrophage Migration Inhibitory Factor, TNF-α—Tumor Necrosis Factor-alpha, TNF-β—Tumor Necrosis Factor-beta, TRAIL—TNF-related apoptosis-inducing ligand, Man—mandible, * *p* < 0.05, *** *p* < 0.0005, **** *p* < 0.0001, ns—not significant.

The concentrations of cytokines IFN-α2 (*p* < 0.0005), IFN-γ (*p* < 0.05), IL-1β (*p* < 0.05), IL-2 (*p* < 0.005), IL-2Rα (*p* < 0.0005), IL-3 (*p* < 0.0005), IL-4 (*p* < 0.05) (Figure 7); IL-5 (*p* < 0.05), IL-6 (*p* < 0.0005), IL-9 (*p* < 0.0001), IL-12(p70) (*p* < 0.005), IL-12(p40) (*p* < 0.005), and IL-15 (*p* < 0.05) (Figure 8) were significantly higher in the mandible periosteum of the study group compared to the control. The concentrations of IL-16 (*p* < 0.05), IL-17 (*p* < 0.05), MIF (*p* < 0.0001), TNF-α (*p* < 0.0005), TNF-β (*p* < 0.0001), and TRAIL (*p* < 0.0001) were significantly higher in the mandible periosteum of the experimental group than in the control group (Figure 9).

The concentrations of IL-1α IL-1ra, IL-7, IL-10, IL-13, and IL-18 did not differ significantly between the control and study groups (Figure 7, Figure 8 and Figure 9).

**Figure 10:**
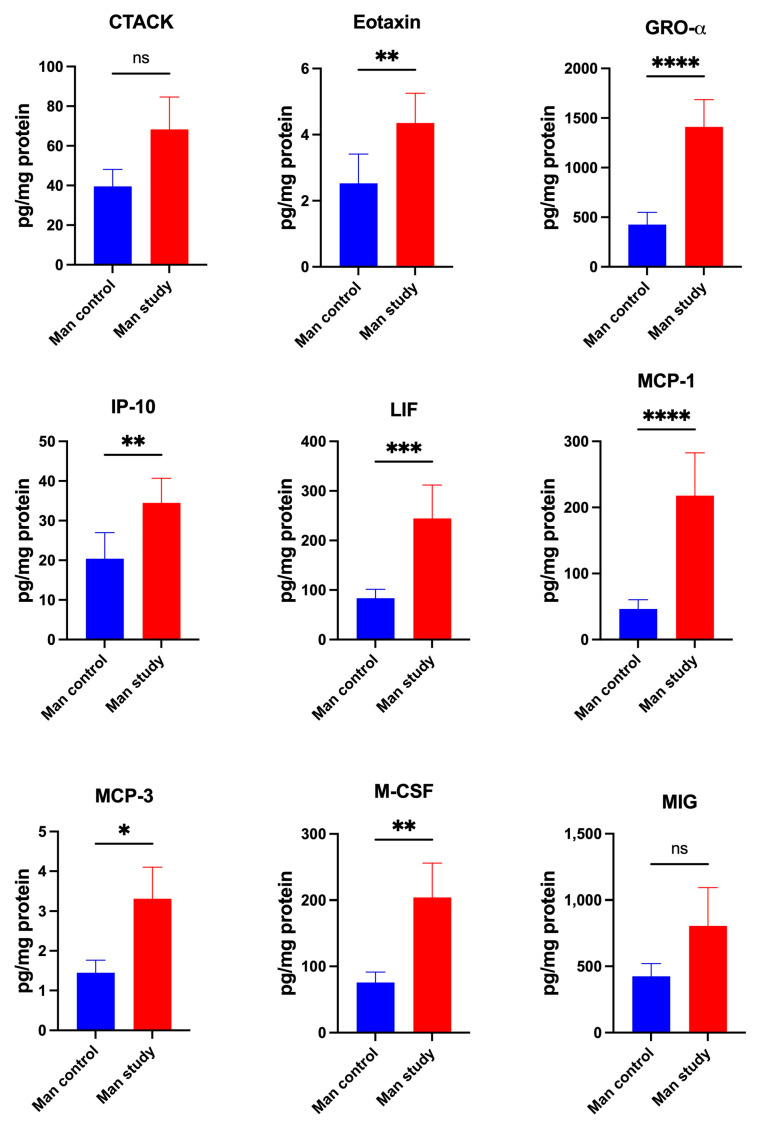
Concentrations of the chemokines in mandibular periosteum: CTACK—Cutaneous T Cell-Attracting Chemokine, GRO-α—Growth-Regulated Oncogene-alpha, IP-10—Interferon-Inducible Protein-10, LIF—Leukemia Inhibitory Factor, MCP-1—Monocyte Chemoattractant Protein-1, MCP-3—Monocyte Chemoattractant Protein-3, M-CSF—Macrophage Colony-Stimulating Factor, MIG—Monokine Induced by Gamma Interferon, Man—mandible, * *p* < 0.05, ** *p* < 0.005, *** *p* < 0.0005, **** *p* < 0.0001, ns—not significant.

**Figure 11:**
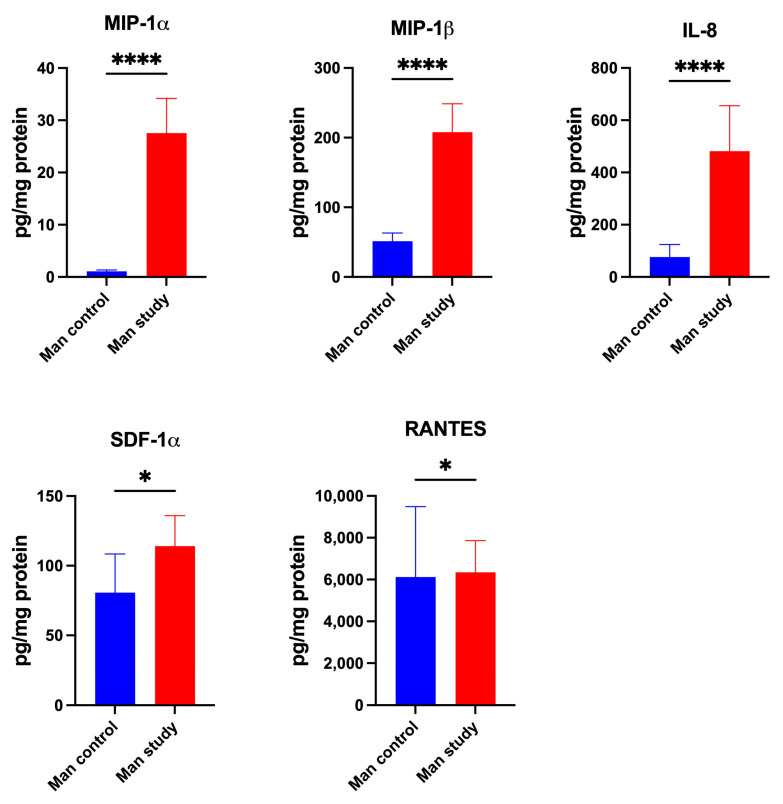
Concentrations of the chemokines in mandibular periosteum: MIP-1α—Macrophage Inflammatory Protein-1 alpha, MIP-1β—Macrophage Inflammatory Protein-1 beta, IL-8—Interleukin-8, SDF-1α—Stromal Cell-Derived Factor-1alpha, RANTES—Regulated on Activation, Normal T Cell Expressed and Secreted, Man—mandible, * *p* < 0.05, **** *p* < 0.00001.

Study group patients were characterized by significantly higher concentrations of chemokines Eotaxin (*p* < 0.005), GRO-α (*p* < 0.0001), IP-10 (*p* < 0.005), LIF (*p* < 0.0005), MCP-1 (*p* < 0.0001), MCP-3 (*p* < 0.05) and M-CSF (*p* < 0.005) (Figure 10); MIP-1α (*p* < 0.0001), MIP-1β (*p* < 0.0001), IL-8 (*p* < 0.0001), SDF-1α (*p* < 0.05), and RANTES (*p* < 0.05) in the mandibular periosteum than patients of control group (Figure 11). The concentrations of CTACK and MIG in the periosteum of mandible were similar in both groups (Figure 10).

**Figure 12:**
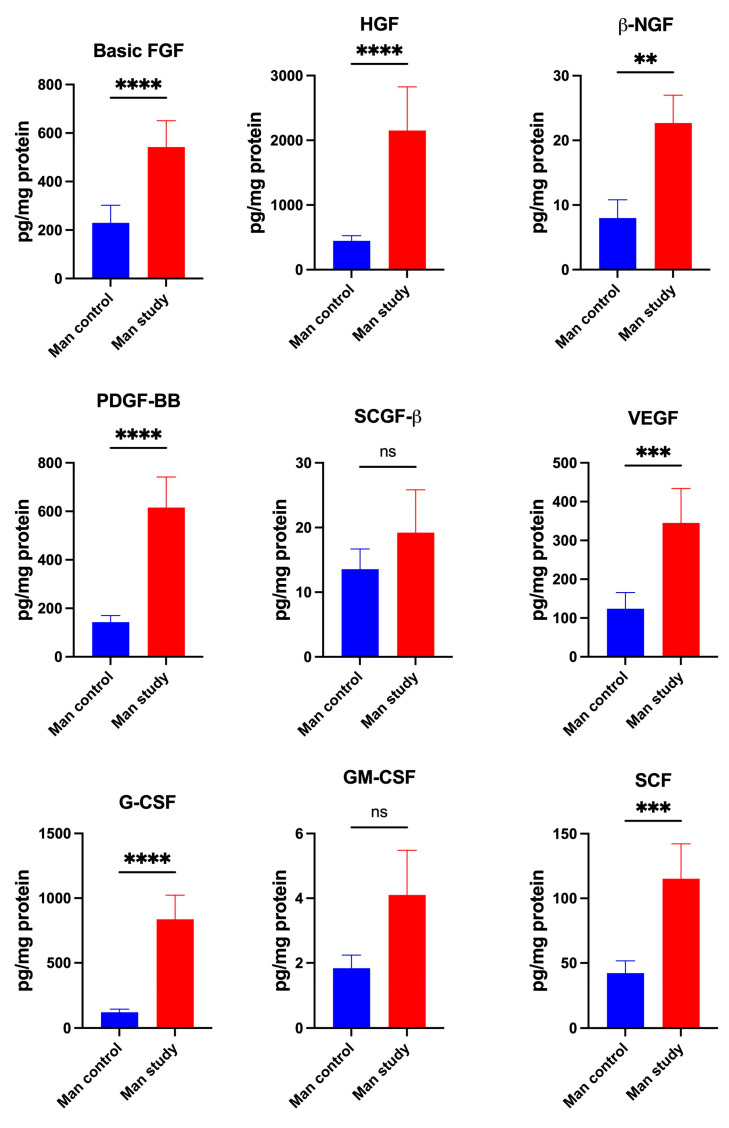
Concentrations of the growth factors in mandibular periosteum: Basic FGF—Basic Fibroblast Growth Factor, HGF—*Hepatocyte Growth Factor*, β-NGF—Nerve Growth Factor-beta, PDGF-BB—Platelet Derived Growth Factor-BB, SCGF-β—Stem Cell Growth Factor-beta, VEGF—Vascular Endothelial Growth Factor, G-CSF—granulocyte colony stimulating factor, GM-CSF—granulocyte macrophage-colony stimulating factor, SCF—Stem Cell Factor, Man—mandible, ** *p* < 0.005, *** *p* < 0.0005, **** *p* < 0.00001, ns—not significant.

The levels of growth factors Basic FGF (*p* < 0.0001), HGF (*p* < 0.0001), β-NGF (*p* < 0.005), PDGF-BB (*p* < 0.0001), VEGF (*p* < 0.0005), G-CSF (*p* < 0.0005), and SCF (*p* < 0.0005) were significantly higher in the periosteum of mandible of the patients in the study group compared to the control (Figure 12). The concentrations of SCGF-β and GM-CSF did not differ significantly between the control and study groups (Figure 12).

## 4. Discussion

The immune system interaction and inflammatory state require a well-coordinated and biochemically active process to restore tissue balance, which will ultimately lead to a soft-tissue wound healing and union of the bone fragments [9]. Cytokines, chemokines, and growth factors are important regulators of cell migration and growth and, as such, are involved in the interaction between surrounding tissue and foreign material [9]. This host reaction to titanium implants is therefore a consequence of the implantation of the biomaterial, leading to the formation of a new bone and the phenomenon of osseointegration [9,10]. From the clinical point of view, a 5–6 month period of bone fragment union with the concurrent use of titanium fixations is considered sufficient to achieve bone union, which should additionally be reflected in the restoration of immunological homeostasis [3,10]. In the case of orthognathic procedures (bimaxillary osteotomy), the positioning of osteotomy segments and muscle attachments, as well as their direction of action, changes. Therefore, bone fixations (titanium miniplates and screws) are kept in place for a longer period, up to 11 months, until the completion of bone remodeling [4]. For the first time, we have demonstrated that, after the clinical and radiological healing of bone fragments with the concurrent use of titanium miniplates and screws (Ti6Al4V alloy) employed for bone fragment fixation purposes, the immune system is still being stimulated in the periosteum of maxilla and mandible. This study outcome has proven that proinflammatory cytokines, chemokines, and some growth factors are released in higher amounts from the periosteum covering the titanium implants in the mandible than from periosteal cells surrounding titanium implants in the maxilla.

In this study, we have used the Bio-Plex Pro assay for measuring cytokine levels from cells exposed to titanium implants. Interpreting that amount of data is challenging because cytokines may have diverse effects on various cell types. Therefore, a common strategy is to further investigate proinflammatory cytokines, as these cytokines appear to play an important role in inflammatory responses toward the implant and may influence the clinical outcome.

Significantly higher levels of IFN-α2, INF-γ, IL-1β, IL-2, IL-2Rα, IL-3, IL-4, IL-5, IL-6, IL-9, IL-12, IL-15, IL-16, IL-17, MIF, TNF-α, TNF-β, and SCF were detected in the homogenate of mandibular periosteal cells exposed to titanium fixations as compared to the control (in maxillary periosteal cells we only observed an increase in IL-1RA, IL-3, IL-9, IL-12(p40), TNF-β, MIF, and SCF). The results of in vitro studies show that macrophages are responsible for the release of the above cytokines that are in contact with a titanium implant during the wound healing phase, approximately up to 3 months after open reduction and osteosynthesis [9]. And, during that period, this is a desirable process because it is part of the connection between bone resorption and its formation, which determines the bone remodeling process [9]. The periosteum samples used in the current research model were collected 11 months after fixation, so these results suggest a prolonged, chronic inflammatory process of the periosteum around the titanium implants. In the case of our patients, it was a process without clinical symptoms, and they were young, generally healthy, well-nourished individuals. It should be emphasized that the prolonged action of the mentioned pro-inflammatory cytokines has been described as a cause of implant rejection, especially in the case of stress-associated stimuli, chronic or acute systemic disease, and stimulus treatment [11]. For example, chronically elevated IL-1β levels are strongly related to type I collagen breakdown in bone and osteoclasts activation and increase the secretion of prostaglandin E2, which may play an essential role in the process of hard-tissue breakdown [12]. Similarly, TNF-α directly increases osteoclasts precursors in bone marrow, indirectly enhances bone loss through the impact on the osteprotegerin/RANKL system, and stimulates the synthesis of prostaglandins and the secretion of proteases by osteoblasts and fibroblasts [12,13]. Those two cytokines are considered a marker of osseointegration complications due to their increased concentration in peri-implant cervicular fluid in the event of implant loss [12,13].

In the situation of increased concentration of so many pro-inflammatory cytokines, especially in the case of homogenate obtained from the periosteum of the mandible, the lack of protective effect of the anti-inflammatory cytokines, including IL-10, the concentration of which does not differ between the test groups and their control reference, is very hazardous. IL-10 suppresses the inflammatory feedback loop that reduces the release of inflammatory mediators as well as downregulates macrophage and Th17 responses by inhibiting the production of pro-inflammatory cytokines including IL-6 and TNF-α [14,15].

In our study, in response to contact with the titanium miniplates and screws, the secretion by cells of the periosteum of growth factors and chemokines followed the same pattern as the secretion of the inflammatory cytokines, especially when it came to the differences between the periosteum of the maxilla and mandible. As in the case of pro-inflammatory cytokines, more changes in the concentration of growth factors and chemokines were observed in the periosteum of the mandible than in the maxilla. It seems that after the 11-month period of exposure to titanium, the increase in the concentration of the chemokines under consideration seems to testify to the directed migration of inflammatory cells during the ongoing chronic inflammatory process. In the homogenates of both facial bones, the mandible and maxilla, we observed a significant increase in the release of chemokines inducing the chemotactic activity of neutrophils (in the maxilla and mandible: MCP-1; in the maxilla: CTAC; in the mandible: Eotaxin, LIF, MCP-3, and RANTES) as well as an increase in the release of chemokines triggering the chemotactic activity of monocytes and neutrophils (in the maxilla and mandible: MIP-1α, MIP-1β, GRO-α, SDF-1α, in the maxilla: MIG, in the mandible: IL-8, IP-10). The observed changes in chemokine concentrations may also suggest an increase in the number or life span of osteoclasts, along with a decrease in the formation and/or life span of osteoblasts [16]. The evidence shows that, for example, MCP-1 initiates osteoblast differentiation, recruits osteoclast precursors, and promotes osteoclast genesis, while MIP-1α augments the differentiation of osteoclasts and inhibits the differentiation of osteoblasts [16]. Similarly, RANTES, through the induction of osteoclast migration, increased resorptive activity, adhesion, and survival time, appears to have a negative impact on bone healing processes at the interface with the titanium implant [16].

It appears that a positive outcome, from the perspective of bone homeostasis, is the observed higher level of HGF in the homogenates of the mandibular and maxillary periosteum exposed to Ti6Al4V. Studies have shown that implants coated with that growth factor exhibit better integration, and the surrounding tissues heal without inflammatory complications [17]. We have also noticed that some cytokines, chemokines, and growth factors (such as G-CSF, IL-3, IL-9, PDGF-BB, and VEGF) are upregulated in the periosteum of both facial bones in the study group as compared to the control, suggesting that they may play a potential role in the process of vascular development. This is a beneficial phenomenon because it accelerates bone healing and ensures proper oxygen and nutrient supply to the bone as well as promotes proper healing and tissue homeostasis [18].

The currently conducted assays do not directly explain the cause of the observed changes in the behavior of the immune system components in the periosteal cells surrounding titanium implants. The Energy Disperse X-Ray Spectroscopy conducted in the earlier studies showed a significantly higher level of titanium, aluminum, and vanadium on the mandibular periosteum surface in the patients treated with titanium miniplates and screws as compared to the control group (around a year after the fixation) [1]. Furthermore, we have also demonstrated that after 11 months following the bone fragment fixation, the content of the aforementioned metal ions was significantly higher in the mandibular periosteum than in the maxillary periosteum surrounding the titanium fixations. We attribute this to the presence of greater forces and stresses acting on the titanium implants in the mobile mandible as compared to the maxilla (work in progress). This issue requires further research. The presence of titanium ions in the periosteum surrounding the implants is explained by the phenomenon of metallosis as referred to in the introduction as well as the wear-and-tear and friction processes (corrosion) observed on the surface of titanium screws and plates [1,19,20]. Titanium causes activation of macrophages directly or after phagocytosis, and the activated macrophages secrete cytokines and chemokines that disrupt bone homeostasis towards the increased osteoclastic bone resorption, as previously mentioned [21]. The studies conducted by Cadosch et al. have shown that titanium ions released due to biocorrosion play a role in recruiting osteoclast precursors at the bone–implant interface by inducing the expression and secretion of macrophage-derived chemokines CCL17 and CCL22 and upregulating the CCR4 receptor [21]. Moreover, titanium enhances the expression of M-CSF and TNF-α, which is confirmed in the periosteal cells of the mandibles of our patients. The evidence has shown that M-CSF as well as TNF α stimulate the differentiation and maturation of osteoclast precursors. In vitro studies have shown that titanium particles induce apoptosis of osteoblasts, which may be due to the increases in TRAIL and TNF-β concentrations, demonstrated by us, in the periosteum of both the maxilla and mandible, after 11 months of exposure to titanium implants [22]. Despite the fact that the phenomenon of apoptosis ensures proper tissue homeostasis, it allows the maintenance of the balance between cell proliferation and death, unfortunately, as the research has shown, that titanium-derived apoptosis inhibits bone formation around the implant [22].

A strength of our study is the inclusion of a homogeneous group of generally healthy patients aged 21 to 30. In this age range, bone remodeling processes (bone formation and resorption) are balanced. Additionally, we examined a large group of cytokines, chemokines, and growth factors associated with the body’s immune response to the presence of titanium fixations. We demonstrated the presence of immune imbalance in the periosteum covering the titanium fixations in both the maxilla and mandible, which, from a future perspective, suggests that consideration should be given to removing titanium miniplates and screws once the bone healing period is complete. This may also indicate the need to improve the quality of the miniplates and screws currently used for bone osteosynthesis or to develop new fixation systems, such as those made from biodegradable materials.

A limitation of this study arises from the small number of patients participating in the research as well as the fact that not all of the components of the immune system have been assayed but only the selected ones.

## 5. Conclusions

In the situation of prolonged exposure (several months after the bone fragment union) to the influence of titanium miniplates and screws, we observe a lack of immunological homeostasis in the periosteal cells of the mandible and maxilla.

Proinflammatory cytokines, chemokines, and some growth factors are released in higher amounts from the periosteum covering titanium implants in the mandible than from the periosteal cells surrounding titanium implants in the maxilla after the 11-month exposure period.

The disturbances in the immunological balance observed in this study should prompt consideration of the decision to remove titanium miniplates and screws after the bone fragments have healed.

## Data Availability

The raw data supporting the conclusions of this article will be made available by the authors, without undue reservation.
